# Psychological Interventions for Prenatal Anxiety in Latinas and Black Women: A Scoping Review and Recommendations

**DOI:** 10.3389/fpsyt.2022.820343

**Published:** 2022-03-15

**Authors:** Carolyn Ponting, Guido G. Urizar, Christine Dunkel Schetter

**Affiliations:** ^1^Department of Psychology, University of California, Los Angeles, Los Angeles, CA, United States; ^2^Department of Psychiatry and Behavioral Sciences, University of California, San Francisco, San Francisco, CA, United States; ^3^Department of Psychology, California State University, Long Beach, Long Beach, CA, United States

**Keywords:** prenatal mental health, anxiety, intervention, Black/African American Latinx/Latina, scoping review

## Abstract

Anxiety symptoms are common among pregnant women worldwide. In the United States, prenatal anxiety symptoms tend to be elevated among Black and Latin American women as compared to non-Latina White women. Despite the high prevalence of anxiety and associations with adverse maternal and offspring outcomes, interventions have not been developed or tailored sufficiently to Black women or Latinas who need efficacious treatment. This article provides a scoping review of articles published since 2017 that test the effects of randomized and non-randomized psychological interventions designed to reduce prenatal anxiety in samples comprised primarily of ethnic/racial minority women. We also review published protocols of planned psychological interventions to reduce prenatal anxiety in order to highlight novel approaches. In addition to summarizing intervention efficacy and participant acceptability, we highlight gaps in the literature which, if addressed, could improve perinatal mental health equity. Finally, we discuss future directions in prenatal anxiety intervention science beginning preconception including intervention design and prevention models.

Anxiety is the most prevalent psychological disorder and it disproportionately affects women. The lifetime prevalence of anxiety disorders in adult women residing in the United States is 40% (1). During pregnancy, pooled prevalence rates indicate that about 15% of women will meet diagnostic criteria for an anxiety disorder (2). Self-reported prenatal anxiety symptoms differ by race and ethnicity. In particular, approximately one third of Black women and Latinas in the U.S. experience elevated anxiety symptoms (3) due in part to structural oppression, which increases financial, relationship, and discrimination related stress in general and during pregnancy (4, 5).

Functional impairment resulting from prenatal anxiety can manifest in ways that adversely affect a woman and her pregnancy. For example, pregnant women with anxiety are less likely to adhere to prenatal care recommendations such as abstaining from substance use or engaging in positive health behaviors (6, 7). They may also avoid prenatal healthcare because it can trigger health-related worries (8). Additionally, prenatal anxiety has been associated with adverse health outcomes for mothers and their infants—specifically postpartum depression, shorter gestation, low birthweight, impaired infant neurodevelopment, and behavioral problems later in childhood (9–11). Given its high prevalence and associated long-term health risks for mothers and their children, prenatal anxiety screening and interventions are urgently needed.

Treatments for anxiety in adults include psychotherapy and medication. However, the safety of psychotropic medications in pregnancy continues to be debated. While medications such as benzodiazepines and serotonin reuptake inhibitors have been reliably linked to adverse infant outcomes (e.g., low birth weight, pre-term birth), the magnitude of these risks is not yet established (12, 13). Additionally, in cases where anxiety is severely impairing, providers must weigh whether abstaining from medication might result in symptom exacerbation (14) with negative consequences for mothers and offspring. Thus, while the American College of Obstetricians and Gynecologists (15) maintains that the cost-benefit analysis for medication ought to be made on a case-by-case basis, psychotherapy is widely considered as a first line intervention to reduce prenatal anxiety.

Nonetheless, referrals to psychotherapeutic services in obstetric settings are infrequent (16). Among low-income and ethnic and racial minority women, structural barriers including lack of insurance and discrimination in prenatal health care settings (17) reduce regular attendance at prenatal health appointments (18, 19). Less frequent interactions with prenatal health care providers along with stigma surrounding disclosure of mental health concerns (20) also limit the likelihood of coordinated mental health care among pregnant ethnic and racial minority women. Survey data substantiate disparities in service utilization by women of color. In a representative sample of over 2,100 women who gave birth in California in 2016, Black women and Latinas utilized perinatal mental health services at significantly lower rates than their non-Latina counterparts despite having more internalizing symptoms (21). Thus, for a variety of reasons, psychotherapy is underutilized during pregnancy and particularly among Black women and Latinas.

Compounding barriers to obtaining mental health services in pregnancy is the need for more evidence regarding which interventions are most likely to be effective. For example, Cognitive Behavioral Therapy (CBT) is considered a gold-standard intervention for anxiety in the general population (22), but is infrequently tested in pregnant samples. Results from intervention studies with small sample sizes (most *n*'s <15) have shown mixed effects of CBT on prenatal anxiety. That is, some studies show CBT to be beneficial in reducing prenatal anxiety symptoms (23–25), whereas others show no intervention effects (26, 27).

In RCTs that enroll a significant number of ethnic and racial minorities, investigators infrequently report results by race and ethnicity (28), masking whether existing prenatal interventions are equally effective for Black women and Latinas. In general, testing the relative efficacy of evidence-based psychotherapies across racial/ethnic groups is much needed. Research to date suggests that ethnic and racial minorities show better psychological outcomes when treated with culturally adapted intervention protocols as compared to standard protocols (29). Thus, assessing whether prenatal intervention effects are generalizable to Black women and Latinas is critical, and can help identify particular psychotherapy modalities or styles of delivery in need of cultural tailoring (30). For example, intervention protocols that standardize clinical consultation with case managers to address system-level factors like housing insecurity that contribute to women's prenatal anxiety may enhance the efficacy of prenatal interventions among at-risk populations.

A recent systematic review evaluated the treatment outcome literature among Black or Latina pregnant women who received a psychological intervention to reduce symptoms of depression or anxiety (31). For inclusion in the review at least 75% of the treatment sample had to identify as Black or Latina. The authors found that that only two published studies—both testing interpersonal therapy—have sought to reduce anxious symptoms during pregnancy (31). In both studies, women who were randomized to the intervention group did not fare better than women in the control condition with respect to a reduction in anxiety symptoms. Therefore, results of the review found that none of the delivered psychotherapies for prenatal anxiety met the criteria to be considered evidence-based among Black women or Latinas.

Data from Ponting et al. (31) show that the last identified anxiety intervention tested with a majority Black and Latina sample was published in 2017. In the present article, we provide an update to the Ponting et al. (31) review by searching for psychological interventions reporting on prenatal anxiety efficacy in studies enrolling at least 50% Black women and Latinas from 2017 through 2021. We also review published protocols for prenatal anxiety reduction during this same time to describe recent patterns in intervention science for prenatal anxiety.

We take a scoping review approach that is appropriate for identifying themes and discussing gaps within a content area (32). This method may be especially helpful when the content area has a limited evidence base, as is the case for anxiety interventions in pregnancy. Based on our review, we suggest several directions to improve efficacy and equity in psychotherapy research for prenatal anxiety as yet.

## Method

### Eligibility Criteria

We utilized a scoping review methodology (32) to search for psychological interventions published during the last 5 years (2017–2021) to treat prenatal anxiety in ethnic and racial minorities. The written protocol for this scoping review was adapted from a previously registered protocol of the authors (see: Prospective Register of Ongoing Systematic Reviews ID: CRD42018106228) in three ways. First, only studies treating anxiety were included. Second a smaller proportion of the treatment sample had to identify as Black or Latina (i.e., >50 vs. >75%). Third, published protocols with anxiety outcome measures were included even if they did not yet have efficacy data.

Thus, studies were included if they tested a psychological intervention delivered during pregnancy, and if they reported on a standardized instrument to measure anxiety (e.g., GAD-7; STAI) at pre and post treatment. Samples had to be comprised of at least 50% Black or Latina participants, a standard used in prior perinatal intervention reviews with ethnic and racial minority women (33). Given the dearth of efficacy studies for prenatal anxiety, we also included published intervention protocols for treating anxiety in pregnancy. Intervention protocols describe RCTs currently in progress. Although we cannot yet report on their efficacy, they reflect upcoming advances in prenatal anxiety treatment modality and delivery. Studies on psychopharmacology or complementary and alternative therapies (e.g., massage, yoga) were excluded.

### Search Strategy

The research team consulted with an experienced research librarian to develop search terms for the scoping review (all search syntax is available in Supplement). Five databases were searched: PubMed©, CINAHL©, PsycINFO©, ProQuest Dissertation and Theses AI©, and Web of Science Articles published from January 2017 through September 2021. In total, searches produced 240 studies. Duplicates were identified using Rayaan (34), an online tool, and confirmed by a member of the review team, leaving 191 unique articles.

**Figure 1:**
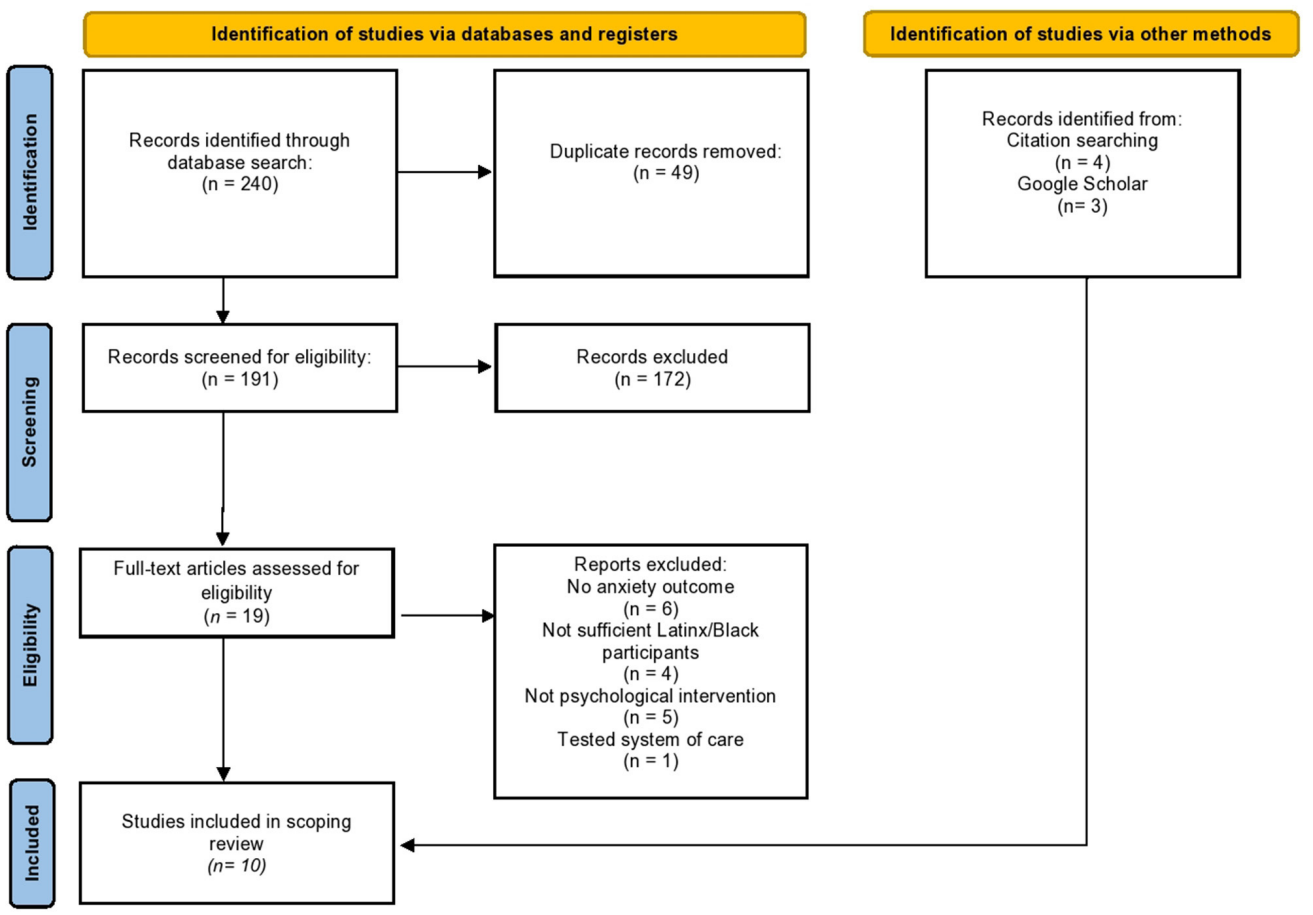
PRISMA scoping review flow diagram for study inclusion.

The methods sections of the 191 articles were examined next. One reviewer screened study abstracts for eligibility based on inclusion/exclusion criteria and consulted with the research team to reach consensus if unsure about inclusion (see Figure 1 for detailed information about exclusion). At this stage, a total of 172 articles were excluded, in most cases because studies did not test a psychological intervention, and instead were observational studies of mental health during pregnancy (*n* = 130). Rayaan (34) was used to store all article abstracts and catalog the reason for inclusion or exclusion. The remaining 19 articles underwent full-text review, during which 16 articles were excluded. Scanning reference sections (e.g., snowball approach), and searching Google Scholar (35) increased search comprehensiveness and led to the addition of seven articles. Thus, a total of seven studies met all inclusion criteria for this scoping review—two were intervention efficacy studies and five were registered intervention protocols for prenatal anxiety.

### Data Extraction

The following were extracted from the two intervention efficacy studies: (1) intervention characteristics (intervention format, treatment modality, provider type, number of sessions, setting); (2) participant demographics (race/ethnicity), (3) U.S. vs. foreign born, and indicators of income); (4) the perinatal period during which the intervention was delivered; (5) type of study design (e.g., RCT, pre-post design); (6) outcomes pertaining to anxiety; (7) the use of cultural adaptations; and (8) intervention acceptability data. The same variables were extracted for studies reporting on intervention protocols (*n* = 5), except for treatment response data given that efficacy data was not yet available. The first author extracted these data and charted them independently using a table approved and tested by the research team; data validity and accuracy were checked by a research assistant.

### Data Quality Assessment

The methodological biases of the empirical treatment studies in this review were assessed using the Cochrane risk of bias assessment. Specifically, six categories of bias are assessed: (a) selection bias, (b) performance bias, (c) detection bias, (d) attrition bias, (e) reporting bias, and (f) baseline imbalance. The protocols for RCTs that are included were not subjected to a data quality assessment for two reasons. First, the randomized nature of the proposed studies indicated low overall biases, and second, outcome data were not yet reported, meaning that bias categories like attrition bias, reporting bias, and baseline imbalances were not able to be assessed. Bias ratings can be found in Table A1.

## Results

### Prenatal Anxiety Intervention Efficacy

Two studies provided data on initial clinical efficacy for prenatal anxiety among Latinas and Black women. Lenze and Potts (36) tested an RCT (*n* = 42) with a majority low-income Black (79%) sample enrolled during their first trimester of pregnancy. The authors reported that women randomized to nine sessions of individual interpersonal psychotherapy for depression delivered by mental health professionals, did not show significant reductions in anxiety symptoms (a secondary outcome). This study was considered to have low methodological bias.

The second study authored by Ruiz et al. (37) used a non-randomized pre-post intervention design (*n* = 15) with a low-income Latina-only sample (31% were foreign born) enrolled during their first trimester of pregnancy. Authors reported that following the receipt of a six-session culturally-adapted combined Acceptance and Commitment Therapy (ACT) and Problem Solving Therapy (PST; delivered by nurse practitioners and midwives), women showed significant reductions in their prenatal anxiety symptoms from baseline to post-intervention. Participants in both studies provided quantitative data about the acceptability of the interventions; scores revealed high levels of satisfaction. This study was considered to have high methodological bias (see Table A1 for bias ratings).

### Registered Protocols for Treating Prenatal Anxiety

For full information on protocol characteristics see Table 1. We identified eight intervention protocols for prenatal anxiety published since 2017. Most protocols (*n* = 6) were RCTs proposing to test the efficacy of CBT. These protocols involved delivery of CBT face-to-face (38, 40, 41, 43), online (42, 45), or in combination with a positive parenting intervention (43). One protocol compared an enhanced *Prenatal Positive Parenting Program with Mellow Bumps*, an intervention focused on promoting antenatal health and mother-infant attachment (44). Face-to-face interventions planned to use hospital or prenatal clinic dissemination settings (38, 40, 41, 43) and delivery models reliant on prenatal nursing professionals (38, 40, 41, 44). Six studies planned to deliver their interventions individually (38, 41, 42), and the other two planned to deliver them in a group setting (43, 44). Three of the five protocols made explicit reference to techniques under consideration to improve cultural fit of their interventions with historically excluded groups of pregnant women (38–40, 43–45), and five planned to collect acceptability data from enrolled women (38–41, 44).

**Table 1:** Design, measurement, hypotheses and results of reviewed studies.

**References**	**Intervention (a) format, (b) treatment modality, (c) # sessions, attendance, (d) provider (and provider education), (e) setting**	**Study design**	**Results main effects**	**Cultural factors**	**Acceptability**
**Efficacy studies**	
Lenz and Potts (36)	(a) individual (b) IPT (c) 9 sessions (one ethnographic introductory session + 8 IPT sessions); plus maintenance treatment session if participant finish all nine sessions (d) Clinical Psychologists, master's level clinicians (e) research clinic, participant homes, or other community locations	RCT	Women in both the intervention and enhanced prenatal care groups did not show significant reductions in anxiety symptoms (STAI-Brief).	Not reported	Participants assigned to brief-IPT reported high scores on the Client Satisfaction Questionnaire at the 37–39 weeks assessment (IPT *M* = 30.60, SD = 1.89, range 25–32)
Ruiz et al. (37)	(a) individual (b) ACT and Problem-Solving Therapy (c) six sessions (d) Nurse practitioners (NPs) or certified nurse midwives (e) primary clinic, OBGYN office	Pilot Pre-Post design	Women in the intervention group showed significant reductions in anxiety symptoms (BAI) between baseline and post-intervention (*d* = 0.31, small to medium effect).	Yes: “considerations for family roles and hierarchies, culturally relevant metaphors/mindfulness exercise”	High satisfaction ratings (*M* = 7 out of 7) for satisfaction, relevance and recommendation to other pregnant people.
**Randomized controlled trial study protocols**					
Atif et al. (38)	(a) individual OR group (b) CBT (c) six sessions, time client dependent ~1 h, option for up to six booster sessions (d) Para-professionals (bachelor's level volunteers with no prior mental health training with nursing backgrounds) (e) hospital	RCT	Primary hypothesis: The intervention will address symptoms of anxiety as measured by the HADS before these become chronic, severe, and debilitating, and therefore will be preventative, allowing women to learn strategies for stress management and problem solving before the symptoms become ingrained.	Yes: “The intervention used culturally appropriate illustrations and examples of healthy activities to set tasks in collaboration with women to encourage engaging unhelpful behaviors”	An initial sample of five women rated the intervention as helpful and acceptable to them and their family members; resulting in greater awareness of their feelings, stress management and strategies to improve wellbeing.
Bright et al. (39)	(a) individual (b) online IPT (c) six 30-min sessions (d) none, self -guided (e) online	RCT	Primary hypothesis: Participants randomized to the intervention will have clinical levels of anxiety at lower percentages post-treatment than women randomized to routine care as measured by the DASS-21.	None stated.	Planned assessment of percentage of participants who report the modules and activities in the intervention as easily understood and navigated to measure acceptability.
Challacombe et al. (40)	(a) individual (b) CBT (c) Fout to five 2-h sessions vs. 8–10 1-h sessions (d) “experienced therapists” (e) national publicly funded healthcare setting	RCT	Primary hypothesis: Participants with clinical diagnoses of OCD, PTSD, Social Anxiety or Panic Disorder randomized to time intensive or standard weekly intervention will show reductions in anxiety as measured by the GAD-7 or disorder specific measures (e.g., OCI for OCD, MI for Panic Disorder, IES for PTSF, SPIN for Social Anxiety).	None stated.	Planned qualitative interviews to assess acceptability of recruitment methods, assessment measures, intervention mode, and delivery.
Jackson et al. (41)	(a) individual (b) CBT (c) eight sessions (d) Perinatal clinical nurse specialist (e) hospital-based perinatal outpatient program	RCT	Primary hypothesis: Participants randomized to the intervention will show less anxiety than those in the control condition, as measured by the STAI. We also hypothesize this study will be feasible in terms of fidelity and deliverability, as well as be highly acceptable to participants.	None stated.	Four open-ended qualitative questions planned to assess acceptability.
Loughnan et al. (42)	(a) individual (b) iCBT (c) three sessions, time client dependent ~1 h (d) None- self-guided (e) client's choice (remote, online)	RCT	Primary hypothesis: Participants randomized to the intervention will show significantly fewer symptoms of anxiety compared to those in usual care, as measured by the GAD-7.	None stated.	–
Melnyk et al. (43)	(a) group (b) CBT + positive parenting (c) six 2-h sessions (d) advanced practice nurses (e) prenatal clinic	RCT	Primary Hypothesis: Participants randomized to the intervention will show significantly less anxiety at 4–6 weeks postpartum (3months post-intervention), and at 6 moths postpartum (8 months post-intervention) than will participants in the attentional control condition as measured by the GAD-7.	Yes: “sessions that are culturally sensitive, readable at the sixth grade reading level and focused on empowering pregnant minority women to engage in healthy lifestyle behaviors”. Prior delivery with ethnic/racial minority women.	–
O'Brien et al. (44)	(a) group (b) Enhanced Triple P for Baby (positive parenting program); Mellow Bumps (reflective functioning, self-care, parenting) (c) four 2-h sessions (ETPB); six 2-h sessions (MB) (d) facilitators with health visiting or midwifery backgrounds (e) multiple	RCT	Primary Hypothesis: Women randomized to either intervention will show reduced anxiety as compared to women receiving usual care.	Yes: Planned interviews with the Heads of Midwifery planned delivery settings to gain further insights into the ways in which the local context and organizational culture might have impacted recruitment	Documented acceptability in high deprivation contexts in prior research studies.
Zuccolo et al. (45)	(a) individual (b) Motherly app (behavioral activation) plus brief online CBT (c) four sessions (d) Psychologists certified in CBT (e) online	RCT	Primary Hypothesis: Participants receiving the Motherly app plus brief online CBT will show significantly greater reduction in symptoms of anxiety (secondary outcome) as compared with participants who receive only psychoeducation in addition to brief online CBT as measured by the GAD-7.	Yes: Treatment targets (e.g., physical activity, nutrition) were specifically selected based on research findings regarding risk factors for individuals from low-income countries. Intervention uses recommendations based on those from Brazilian Ministry of Health. Online delivery of CBT selected due to dearth of human and financial resources in Brazil.	–

*ACT, Acceptance and Commitment Therapy; CBT, Cognitive Behavioral Therapy; iCBT, internet-delivered Cognitive Behavioral Therapy; ETPB, Enhanced Triple P for Baby; IPT, Interpersonal Therapy; MB, Mellow Bumps; RCT, Randomized Control Trail; BAI, Beck Anxiety Inventory; GAD-7, Generalized Anxiety Disorder- 7item; HADS, Hospital Anxiety and Depression Scale; STAI, State-Trait Anxiety Inventory; DASS-21, Depression, Anxiety and Stress Scale-21; OCI, Obsessive Compulsive Inventory-Revised; MI, Mobility Inventory; IES, Impact of Events Scale; SPIN, The Social Phobia Inventory*.

## Discussion

This scoping review sought to update current knowledge in the last 5 years (2017–2021) on psychological intervention efficacy for prenatal anxiety in samples comprised of at least 50% Latinas or Black women. Only two completed and published studies met criteria for inclusion; of those, a non-randomized trial testing an intervention integrating ACT and problem-solving therapy in a small Latina sample (*n* = 15) showed beneficial effects for prenatal anxiety. A prior systematic review without any date restrictions and a higher threshold for ethnic/racial minority inclusion (i.e., 75%) also identified only two trials seeking to reduce prenatal anxiety (31). The present review, using a less stringent inclusion criteria for proportion of Latinas or Black women enrolled, found just one new published intervention trial since Ponting et al. (31). Thus, to date there are a total of three published studies (*total n* = 101)—two in primarily Black samples, one in a Latina only sample—that have sought to treat prenatal anxiety in Latinas or Black women. Meta-analytic data indicate that there are nine additional published RCTs comprised of primarily white women (*total n* = 443), most testing CBT, that show medium effect sizes of psychotherapy on prenatal anxiety (46). Thus, although there are relatively few prenatal anxiety interventions overall, there is evidence of symptom improvement for non-Latina white women, but not as yet for Latinas or Black women. This gap in the literature requires attention by researchers and clinicians.

We also reviewed published protocols for RCTs testing interventions for prenatal anxiety. Despite the identified disparity in efficacious anxiety interventions for Black women and Latinas, the eight protocols identified are encouraging for at least two reasons. First, four of the eight protocols planned to train nurses and midwives who are perinatal health professionals without a mental health background. This is likely to be a promising approach. The feasibility of this delivery model is supported by nurses and midwives' frequent contact with pregnant women and extensive training in pregnancy-specific health information (47). In conjunction with increased funding for home visiting programs for pregnant women under the Affordable Care Act, this workforce is well-positioned to address common pregnancy-specific worries and concerns. Recent meta-analyses find that midwife-delivered psychological interventions are efficacious for reducing perinatal anxiety and depression (48, 49).

Second, three of the protocols (38, 43, 44) identify theoretical mechanisms of treatment change, which can serve as psychosocial targets across psychotherapeutic modalities. For example, the O'Brien et al. (44) protocol stands out because the proposed mediators of change are situated within the developmental context of pregnancy. The enhanced *Triple P for Parenting and Mellow Bumps* protocol is teaching pregnant women and their partners about infant and child development and will test whether improvements in knowledge around infant development will lead to maternal anxiety symptoms. Though psychologists have urged for better measurement of potential mechanisms of intervention that are related to symptom change [e.g., (50)], prenatal intervention work has lagged in this domain. Identifying the psychosocial targets most likely to lead to improved anxiety profiles during pregnancy is a worthy avenue for future investigation.

## Future Considerations to Improve Equity in Prenatal Anxiety Intervention

The disproportionate impact of socio-political stressors on Black women and Latinas in the United States adversely impacts anxiety symptoms among these groups (51), and we lack evidence-based psychotherapies to intervene at the individual level in pregnancy. In this context of high unmet need, we argue that increased attention to family involvement, pregnancy-specific anxiety, and prevention efforts during preconception (52) are likely to improve engagement and clinical outcomes among ethnic and racial minority pregnant women.

Our recommendation is to include a woman's family members as part of perinatal anxiety interventions to increase her comfort and involvement with treatment. We know that pregnant women have significant interest in receiving family support during their prenatal health care (53), yet no published intervention identified in the present systematic review or in past reviews (31, 54) have involved partner or extended family participation. Public health programs have increasingly sought to involve partners in perinatal care given that couples' anxiety symptoms have bidirectional effects in pregnancy (55) and influence early parenting behaviors (30). There is emerging evidence among samples of primarily white women that brief psychological interventions for pregnant women at risk for anxiety incorporating partners or family can successfully reduce symptoms in both women and their family members (56). The benefits of family engagement may be even greater for pregnant Latinas and Black women whose valuing of close family relationships (e.g., familism) is an important cultural source of resilience during pregnancy (51, 57).

Further, it is possible that familial participation may reduce concerns as reported by the pregnant women in several qualitative studies—that seeking help for anxiety might be negatively perceived as prioritizing themselves over their families (53). However, interventions that consider including partners or family members should take into account history of family violence and prioritize safety as well as ensure that women feel comfortable including their family in such interventions.

Recent research draws attention to a range of pregnancy-specific worries that should be addressed in prenatal anxiety interventions (9). Pregnancy-specific anxiety—or concerns specific to a woman's pregnancy, labor and delivery, and future parenting—is estimated to occur in 29% of women in high income countries (58). Yet, intervention protocols for prenatal anxiety often leave out content about labor and delivery, or about common prenatal medical conditions in pregnancy (e.g., gestational diabetes, preeclampsia). In fact, fear of childbirth and pregnancy-specific anxiety have largely been treated separately from other anxiety symptoms or disorders, and are frequently addressed using only standard prenatal health education (59). Integrating prenatal health education and psychotherapy may increase intervention relevance and improve birth and child outcomes, improving care for pregnant women with anxiety.

*MUMentum* (25) is an internet delivered CBT protocol for perinatal depression and anxiety and a good example of integrating psychotherapy and prenatal education. *MUMentum* supplies prenatal education resources for women, including topics like attachment during pregnancy and intrusive thoughts about childbirth. Efficacy data among Australian women show that randomization to the *MUMentum* intervention resulted in medium to large reductions in anxiety (25). Examining how *MUMentum* and other interventions might impact pregnancy-specific anxiety is a worthy next step, as pregnancy-specific anxiety has been linked to length of gestation, low-birthweight, and adverse physical and mental health outcomes for offspring (60). Psychological intervention trials that can better tailor their content to fit specific and prevalent pregnancy-related worries stand to have particular impact on ethnic and racial minority women who are disproportionately likely to report high pregnancy-specific anxiety (6, 61, 62) and encounter adverse birth outcomes (63).

Finally, the preconception period—before a person is pregnant— is increasingly a window of interest for psychological interventions seeking to improve the overall health of women during pregnancy. Addressing preconception anxiety merits consideration, as symptoms have been associated with postpartum anxiety and later mother-infant bonding problems (64). Thus, providing health promotion information and screening for anxiety symptoms during preventive visits might be worthwhile, especially if women are of reproductive age and are seeking to be pregnant. This same strategy can be applied to interconception periods and appears to be an indicated and low burden form of prevention for women with a prior adverse pregnancy outcome [e.g., traumatic delivery, fetal loss; (65)]. Though referral and treatment may improve women's health broadly and set the stage for a healthier pregnancy in the future, equitable outcomes will require that screening and education is culturally responsive, treatment is disseminated outside of health care settings, and that providers acknowledge historical and current reproductive oppression (66).

In conclusion, this scoping review provides a strong basis for empirically testing and culturally tailoring prenatal anxiety interventions to optimize the health of racial and ethnic minority mothers and their infants. Given longstanding disparities between Black and Latina and non-Latina white women in pre- and postnatal health outcomes and in access to preventive interventions, policy changes are a pre-requisite for prenatal mental health equity. Still, at the individual level, community-based psychological intervention studies for Black women and Latinas can help to identify particular skills, knowledge, and connections to community support best suited for regulating prenatal anxiety. The success of these endeavors will depend on researchers' ability to engage with community stakeholders to appropriately address mistrust of intervention research given historical abuses and current inequities in care in minority communities (67). Researchers can assess intervention acceptability among Latinas and Black women and address pregnancy-specific worries or basic needs to increase the relevance of available treatments for prenatal anxiety. Health promotion programs that can build on the strengths of Latinx and Black communities in the preconception period are also warranted to improve intergenerational outcomes.

## Author Contributions

CP conducted the analyses, visualized the results, conceptualized the aims of the review, and wrote the first draft the review. GU contributed to the writing of the article (introduction, discussion) and provided feedback on several drafts. CD assisted with conceptualization of the aims, contributed to the writing, oversaw research administration, and contributed to the framing of the introduction and discussion. All authors contributed to the article and approved the submitted version.

## Conflict of Interest

The authors declare that the research was conducted in the absence of any commercial or financial relationships that could be construed as a potential conflict of interest.

## Publisher's Note

All claims expressed in this article are solely those of the authors and do not necessarily represent those of their affiliated organizations, or those of the publisher, the editors and the reviewers. Any product that may be evaluated in this article, or claim that may be made by its manufacturer, is not guaranteed or endorsed by the publisher.

## Appendix

**Table A1:** Assessment of study bias.

	**Random on sequence generation**	**Allocation concealment**	**Blinding of participants and personnel**	**Blinding of outcome assessment**	**Incomplete outcome data**	**Selective reporting**	**Baseline imbalance**	**Overall Bias**
Lenze and Potts (36)	⊕	⊕	⊕	∅	⊕	⊕	⊕	Low
Ruiz et al. (37)	⊗	⊗	⊗	⊗	∅	⊕	⊕	High

*⊕Indicates low risk of bias, ∅indicates unclear risk of bias, and ⊗indicates high risk of bias. Studies rated as “low risk of bias” on four of the six categories were considered to have an overall low risk of bias; studies with two or three categories rated as “low risk of bias” were considered to have an overall medium risk of bias; and studies with one or fewer categories rated as “low risk of bias” were considered to have an overall high risk of bias*.

## References

[1] Kessler RC Petukhova M Sampson NA Zaslavsky AM Wittchen H-U. Twelve-month and lifetime prevalence and lifetime morbid risk of anxiety and mood disorders in the United States. Int J Methods Psychiatr Res. (2012) 21:169–84. 10.1002/mpr.1359 22865617 PMC4005415

[2] Dennis CL Falah-Hassani K Shiri R. Prevalence of antenatal and postnatal anxiety: systematic review and meta-analysis. Br J Psychiatry. (2017) 210:315–23. 10.1192/bjp.bp.116.187179 28302701

[3] Grobman W Parker C Wadhwa P Willinger M Simhan H Silver B. Racial/Ethnic disparities in measures of self-reported psychosocial states and traits during pregnancy. Am J Perinatol. (2016) 33:1426–32. 10.1055/s-0036-1586510 27500932 PMC5821109

[4] Liu CH Giallo R Doan SN Seidman LJ Tronick E. Racial and ethnic differences in prenatal life stress and postpartum depression symptoms. Arch Psychiatr Nurs. (2016) 30:7–12. 10.1016/j.apnu.2015.11.002 26804495

[5] Rosenthal L Lobel M. Explaining racial disparities in adverse birth outcomes: unique sources of stress for Black American women. Soc Sci Med. (2011) 72:977–83. 10.1016/j.socscimed.2011.01.013 21345565

[6] Arch JJ. Pregnancy-specific anxiety: which women are highest and what are the alcohol-related risks?. Compr Psychiatry. (2013) 54:217–28. 10.1016/j.comppsych.2012.07.010 22943960

[7] Beijers R Buitelaar JK de Weerth C. Mechanisms underlying the effects of prenatal psychosocial stress on child outcomes: beyond the HPA axis. Eur Child Adolesc Psychiatry. (2014) 23:943–56. 10.1007/s00787-014-0566-3 24875898

[8] Prescott J Mackie L Rathbone AL. Predictors of health anxiety during pregnancy. MHealth. (2018) 4:16. 10.21037/mhealth.2018.04.04 29963561 PMC5994463

[9] Dunkel Schetter C Tanner L. Anxiety, depression and stress in pregnancy: implications for mothers, children, research, and practice. Curr Opin Psychiatry. (2012). 10.1097/YCO.0b013e3283503680 22262028 PMC4447112

[10] Phua DY Chen H Chong YS Gluckman PD Broekman BFP Meaney MJ. Network analyses of maternal pre- and post-partum symptoms of depression and anxiety. Front Psychiatry. (2020) 11:785. 10.3389/fpsyt.2020.00785 32848949 PMC7424069

[11] Shahhosseini Z Pourasghar M Khalilian A Salehi F. A review of the effects of anxiety during pregnancy on children's health. Materia Socio Medica. (2015) 27:200–2. 10.5455/msm.2015.27.200-202 26236168 PMC4499279

[12] Huitfeldt A Sundbakk LM Skurtveit S Handal M Nordeng H. Associations of maternal use of benzodiazepines or benzodiazepine-like hypnotics during pregnancy with immediate pregnancy outcomes in Norway. JAMA Network Open. (2020) 3:e205860. 10.1001/jamanetworkopen.2020.5860 32568398 PMC7309438

[13] Marchesi C Ossola P Amerio A Daniel BD Tonna M De Panfilis C. Clinical management of perinatal anxiety disorders: a systematic review. J Affect Disord. (2016) 190:543–50. 10.1016/j.jad.2015.11.004 26571104

[14] Freeman MP. Perinatal depression. JAMA. (2019) 321:550. 10.1001/jama.2018.21247 30747953

[15] American College of Obstetricians Gynecologists (2008). Use of Psychiatric Medications During Pregnancy and Lactation. Available online at: http://unmfm.pbworks.com/w/file/fetch/81072005/pb092.pdf

[16] Leddy MA Lawrence H Schulkin J. Obstetrician-Gynecologists and women's mental health: findings of the collaborative ambulatory research network 2005–2009. Obstet Gynecol Survey. (2011) 66:316–23. 10.1097/OGX.0b013e31822785ee 21794195

[17] Novick G. Women's experience of prenatal care: an integrative review. J Midwif Womens Health. (2009) 54:226–37. 10.1016/j.jmwh.2009.02.003 19410215 PMC2754192

[18] Kozhimannil KB Trinacty CM Adams AS Huskamp HA Busch AB. Racial and ethnic disparities in postpartum depression care among low-income women. Psychiatr Serv. (2014) 62:619–25. 10.1176/appi.ps.62.6.619 21632730 PMC3733216

[19] Lucero NB Beckstrand RL Callister LC Sanchez Birkhead AC. Prevalence of postpartum depression among Hispanic immigrant women. J Am Acad Nurse Pract. (2012) 24:726–34. 10.1111/j.1745-7599.2012.00744.x 23190130

[20] Watson H Harrop D Walton E Young A Soltani H. A systematic review of ethnic minority women's experiences of perinatal mental health conditions and services in Europe. PLoS ONE. (2019) 14:e0210587. 10.1371/journal.pone.0210587 30695019 PMC6351025

[21] Declercq E Feinberg E Belanoff C. Racial inequities in the course of treating perinatal mental health challenges: results from listening to mothers in California. Birth. (2021) 49:132–40. 10.1111/birt.12584 34459012 PMC9292331

[22] Abramowitz JS Deacon BD Whiteside SP. Exposure Therapy for Anxiety: Principles and Practice. 2nd ed. New York, NY: Guilford Press (2019).

[23] Christian LM Storch EA. Cognitive behavioral treatment of postpartum onset. Clin Case Stud. (2009) 8:72–83. 10.1177/1534650108326974

[24] Green SM Haber E Frey BN McCabe RE. Cognitive-behavioral group treatment for perinatal anxiety: a pilot study. Arch Womens Mental Health. (2015) 18:631–8. 10.1007/s00737-015-0498-z 25652951

[25] Loughnan SA Sie A Hobbs MJ Joubert AE Smith J Haskelberg H. A randomized controlled trial of ‘MUMentum Pregnancy': internet-delivered cognitive behavioral therapy program for antenatal anxiety and depression. J Affect Disord. (2019) 243:381–90. 10.1016/j.jad.2018.09.057 30266030

[26] Bittner A Peukert J Zimmermann C Junge-Hoffmeister J Parker LS Stöbel-Richter Y. Early intervention in pregnant women withelevated anxiety and depressive symptoms. J Perinat Neonat Nurs. (2014) 28:185–95. 10.1097/JPN.0000000000000027 25062520

[27] Salehi F Pourasghar M Khalilian A Shahhosseini Z. Comparison of group cognitive behavioral therapy and interactive lectures in reducing anxiety during pregnancy: a quasi experimental trial. Medicine. (2016) 95:e5224. 10.1097/MD.0000000000005224 27787386 PMC5089115

[28] Polo A Makol B Castro A Polo AJ Makol BA Castro AS. Diversity in randomized clinical trials of depression: a 36-year review. Clin Psychol Rev. (2018) 67:22–35. 10.1016/j.cpr.2018.09.004 30292439

[29] Benish SG Quintana S Wampold BE. Culturally adapted psychotherapy and the legitimacy of myth: a direct-comparison meta-analysis. J Couns Psychol. (2011) 58:279–89. 10.1037/a0023626 21604860

[30] Fisher JA Kalbaugh CA. Challenging assumptions about minority participation in US clinical research. Am J Public Health. (2011) 101:2217–22. 10.2105/AJPH.2011.300279 22021285 PMC3222419

[31] Ponting C Mahrer NE Zelcer H Dunkel Schetter C Chavira DA. Psychological interventions for depression and anxiety in pregnant Latina and Black women in the United States: a systematic review. Clin Psychol Psychother. (2020) 27:249–65. 10.1002/cpp.2424 31960525 PMC7125032

[32] Munn Z Peters MD Stern C Tufanaru C McArthur A Aromataris E. Systematic review or scoping review? Guidance for authors when choosing between a systematic or scoping review approach. BMC Med Res Methodol. (2018) 18:1–7. 10.1186/s12874-018-0611-x 30453902 PMC6245623

[33] Lara-Cinisomo S Ramirez Olarte A Rosales M Barrera AZ. A systematic review of technology-based prevention and treatment interventions for perinatal depression and anxiety in latina and African American women. Matern Child Health J. (2021) 25:268–81. 10.1007/s10995-020-03028-9 33389589

[34] Ouzzani M Hammady H Fedorowicz Z Elmagarmid A. Rayyan–a web and mobile app for systematic reviews. Syst Rev. 5:210 (2016). 10.1186/s13643-016-0384-4 27919275 PMC5139140

[35] Gusenbauer M Haddaway NR. Which academic search systems are suitable for systematic reviews or meta-analyses? Evaluating retrieval qualities of Google Scholar, PubMed, and 26 other resources. Res Synth Methods. (2020) 11:181–217. 10.1002/jrsm.1378 31614060 PMC7079055

[36] Lenze SN Potts MA. Brief Interpersonal Psychotherapy for depression during pregnancy in a low-income population: a randomized controlled trial. J Affect Disord. (2017) 210:151–7. 10.1016/j.jad.2016.12.029 28038377 PMC5292056

[37] Ruiz RJ Newman M Records K Wommack JC Stowe RP Pasillas RM. Pilot study of the mastery lifestyle intervention. Nurs Res. (2019) 68:494–500. 10.1097/NNR.0000000000000384 31693556

[38] Atif N Nazir H Zafar S Chaudhri R Atiq M Mullany LC. Development of a psychological intervention to address anxiety during pregnancy in a low-income country. Front Psychiatry. (2020) 10:1–13. 10.3389/fpsyt.2019.00927 31998151 PMC6967413

[39] Bright KS Mughal MK Wajid A Lane-Smith M Murray L Roy N. Internet-based interpersonal psychotherapy for stress, anxiety, and depression in prenatal women: study protocol for a pilot randomized controlled trial. Trials. (2019) 20:814. 10.1186/s13063-019-3897-z 31888712 PMC6938015

[40] Challacombe FL Potts L Carter B Lawrence V Husbands A Howard LM. Optimising psychological treatment for Anxiety DisordErs in Pregnancy (ADEPT): study protocol for a feasibility trial of time-intensive CBT versus weekly CBT. Pilot Feasib Stud. (2021) 7:101. 10.1186/s40814-021-00838-8 33931111 PMC8085465

[41] Jackson KT Parkinson S Jackson B Mantler T. Examining the impact of trauma-informed cognitive behavioral therapy on perinatal mental health outcomes among survivors of intimate partner violence (the PATH study): Protocol for a feasibility study. JMIR Res Protoc. (2018) 20:e134. 10.2196/resprot.9820 29802091 PMC5993975

[42] Loughnan SA Newby JM Haskelberg H Mahoney A Kladnitski N Smith J. Internet-based cognitive behavioural therapy (iCBT) for perinatal anxiety and depression versus treatment as usual: study protocol for two randomised controlled trials. Trials 2018. (2018) 19:1–11. 10.1186/s13063-017-2422-5 29357918 PMC5778736

[43] Melnyk BM Gennaro S Szalacha LA Hoying J O'Connor C Cooper A. Randomized controlled trial of the COPE-P intervention to improve mental health, healthy lifestyle behaviors, birth and post-natal outcomes of minority pregnant women: study protocol with implications. Contemp Clin Trials. (2020) 98:106090. 10.1016/j.cct.2020.106090 32745703 PMC7686149

[44] O'Brien R Buston K Wight D Mcgee E White J Henderson M. A realist process evaluation of Enhanced Triple P For Baby And Mellow Bumps, Within A Trial Of Healthy Relationship Initiatives For The Very Early Years (THRIVE): study protocol for a randomized controlled trial. Trials. (2019) 20:351. 10.1186/s13063-019-3395-3 31196169 PMC6567913

[45] Zuccolo PF Xavier MO Matijasevich A Polanczyk G Fatori D. A smartphone-assisted brief online cognitive-behavioral intervention for pregnant women with depression: a study protocol of a randomized controlled trial. Trials. (2021) 22:227. 10.1186/s13063-021-05179-8 33757591 PMC7985923

[46] Li C Sun X Li Q Sun Q Wu B Duan D. Role of psychotherapy on antenatal depression, anxiety, and maternal quality of life: a meta-analysis. Medicine. (2020) 99:e20947. 10.1097/MD.0000000000020947 32629701 PMC7337511

[47] Marzalik PR Feltham KJ Jefferson K Pekin K. Midwifery education in the U.S. - certified nurse-midwife, certified midwife and certified professional midwife. Midwifery. (2018) 60:9–12. 10.1016/j.midw.2018.01.020 29471175

[48] Han Q Guo M Ren F Duan D Xu X. Role of midwife-supported psychotherapy on antenatal depression, anxiety and maternal health: a meta-analysis and literature review. Exp Ther Med. (2020) 20:2599. 10.3892/etm.2020.9011 32765754 PMC7401497

[49] Wang TH Pai LW Tzeng YL Yeh TP Teng YK. Effectiveness of nurses and midwives-led psychological interventions on reducing depression symptoms in the perinatal period: a systematic review and meta-analysis. Nurs Open. (2021) 8:2117–30. 10.1002/nop2.764 33452740 PMC8363390

[50] Kazdin AE. Mediators and mechanisms of change in psychotherapy research. Annu Rev Clin Psychol. (2007) 3:1–27. 10.1146/annurev.clinpsy.3.022806.091432 17716046

[51] Sumbul T Spellen S McLemore MR. A transdisciplinary conceptual framework of contextualized resilience for reducing adverse birth outcomes. Qual Health Res. (2020) 30:105–18. 10.1177/1049732319885369 31752598

[52] Howard LM Khalifeh H. Perinatal mental health: a review of progress and challenges. World Psychiatry. (2020) 19:313–27. 10.1002/wps.20769 32931106 PMC7491613

[53] O'Mahen H Fedock G Henshaw E Himle JA Forman J Flynn HA. Modifying CBT for perinatal depression: what do women want? A qualitative study. Cogn Behav Pract. (2012) 19:359–71. 10.1016/j.cbpra.2011.05.005

[54] Evans K Spiby H Morrell JC. Non-pharmacological interventions to reduce the symptoms of mild to moderate anxiety in pregnant women. A systematic review and narrative synthesis of women's views on the acceptability of and satisfaction with interventions. Arch Womens Ment Health. (2020) 23:11–28. 10.1007/s00737-018-0936-9 30613846 PMC6987064

[55] MajdandŽi ć M de Vente W Feinberg ME Aktar E Bögels SM. Bidirectional associations between coparenting relations and family member anxiety: a Review and conceptual model. Clin Child Fam Psychol Rev. (2012) 15:28–42. 10.1007/s10567-011-0103-6 22124791 PMC3282913

[56] Thome M Arnardottir SB. Evaluation of a family nursing intervention for distressed pregnant women and their partners: a single group before and after study. J Adv Nurs. (2013) 69:805–16. 10.1111/j.1365-2648.2012.06063.x 22709258

[57] Campos B Schetter CD Abdou CM Hobel CJ Glynn LM Sandman CA. Familialism, social support, and stress: positive implications for pregnant Latinas. Cult Divers Ethnic Minority Psychol. (2008) 14:155–62. 10.1037/1099-9809.14.2.155 18426288 PMC2859297

[58] Chandra PS Nanjundaswamy MH. Pregnancy specific anxiety: an under-recognized problem. World Psychiatry. (2020) 19:336–7. 10.1002/wps.20781 32931120 PMC7491640

[59] Stoll K Swift EM Fairbrother N Nethery E Janssen P. A systematic review of nonpharmacological prenatal interventions for pregnancy-specific anxiety and fear of childbirth. Birth. (2018) 45:7–18. 10.1111/birt.12316 29057487

[60] Dunkel Schetter C. Psychological science on pregnancy: stress processes, biopsychosocial models, and emerging research issues. Annu Rev Psychol. (2011) 62:531–58. 10.1146/annurev.psych.031809.130727 21126184

[61] Mancuso RA Schetter CD Rini CM Roesch SC Hobel CJ. Maternal prenatal anxiety and corticotropin-releasing hormone associated with timing of delivery. Psychosom Med. (2004) 66:762–9. 10.1097/01.psy.0000138284.70670.d5 15385704

[62] Ramos IF Guardino CM Mansolf M Glynn LM Sandman CA Hobel CJ. Pregnancy anxiety predicts shorter gestation in Latina and non- Latina white women: the role of placental corticotrophin-releasing hormone. Psychoneuroendocrinology. (2019) 99:166–73. 10.1016/j.psyneuen.2018.09.008 30245329 PMC6231951

[63] Almeida AF Rocha NP Silva AG. Methodological quality of manuscripts reporting on the usability of mobile applications for pain assessment and management: a systematic review. Int J Environ Res Public Health. (2020) 17:785. 10.3390/ijerph17030785 32012674 PMC7038093

[64] Olsson CA Spry EA Alway Y Moreno-Betancur M Youssef G Greenwood C. Preconception depression and anxiety symptoms and maternal-infant bonding: a 20-year intergenerational cohort study. Arch Womens Mental Health. (2020) 24:513–23. 10.1007/s00737-020-01081-5 33111170

[65] Johnson K Atrash H Johnson A. Policy and finance for preconception care: opportunities for today and the future. Womens Health Issues. (2008) 18:S2–9. 10.1016/j.whi.2008.09.006 19059547

[66] Dehlendorf C Akers AY Borrero S Callegari LS Cadena D Gomez AM. Evolving the preconception health framework: a call for reproductive and sexual health equity. Obstet Gynecol. (2021) 137:234. 10.1097/AOG.0000000000004255 33416289 PMC7813442

[67] Kuhlman KR Urizar Jr GG Robles TF Yim IS Schetter CD. Testing plausible biopsychosocial models in diverse community samples: common pitfalls and strategies. Psychoneuroendocrinology. (2019) 107:191–200. 10.1016/j.psyneuen.2019.05.017 31150964 PMC6635037

