# Supplementary material for: Psychological Interventions for Prenatal Anxiety in Latinas and Black Women: A Scoping Review and Recommendations
Source: Front Psychiatry. 2022 Mar 15;13:820343. doi: 10.3389/fpsyt.2022.820343 (PMC8965279; doi:10.3389/fpsyt.2022.820343)
Supplement: Supplementary file 1 [file Image_1.pdf]

Supplementary File: Search Terms

Psych Info:
((prenatal OR antenatal OR pregnancy) AND (intervention OR treatment OR therapy) AND (anxiety) , AND (African-American OR Black OR Latino OR Hispanic OR minority))

33 results

CINAHL
(prenatal OR antenatal OR pregnancy) AND (intervention OR treatment OR therapy) AND (anxiety), AND (African-American OR Black OR Latino(a) OR Hispanic OR minority)
Limiters - Published Date: 20170101-20211231
Expanders - Apply equivalent subjects
Search modes - Boolean/Phrase

29 results

Web of Science
(TS= (prenatal OR antenatal OR pregnancy) AND TI= (intervention OR treatment OR therapy) AND TS=(anxiety) AND TS=(African-American OR Black OR Latino OR Hispanic OR minority))

14 results

ProQuest Dissertation and Theses AI:
((prenatal OR antenatal OR pregnancy) AND (intervention OR treatment OR therapy) AND (anxiety) AND (African-American OR Black OR Latino OR Hispanic OR minority))

20 results

PubMed
(prenatal OR antenatal OR pregnancy) [tiab] AND (intervention OR treatment OR therapy) [tiab] AND (anxiety)[tiab] AND (African-American OR Black OR Latino(a) OR Hispanic OR minority) [tiab]

101 articles
